# 

**Published:** 1896-04

**Authors:** 


					﻿Southern Medical Record.
A Monthly Journal of Medicine and Surgery.
Vol. XXVI. ~
ATLANTA, GA, APRIL, 1896.
No. IV.^
BERNARD WOLFF, M. D, Editor.
LOUIS H. JONES, M. D, Business Manager.
Associate Editors:
A. W. GRIGGS, M. D.
WILLIS F. WESTMORELAND, M. D.
j. McFadden gastón, m. d.
Entered at the Post Office at Atlanta, Ga., as Second-Class Matter.
The Foote & Davies Co., Atlanta, Ga.
				

